# Psychiatry Meets Cardiology: A Case Report on the Need for Mental Health Assessment in the Evaluation of Cardiovascular Symptoms

**DOI:** 10.1155/2022/5415196

**Published:** 2022-04-21

**Authors:** Kofi Tekyi Asamoah, Richard Dei-Asamoa

**Affiliations:** ^1^National Cardiothoracic Centre, Korle-Bu Teaching Hospital, Accra, Ghana; ^2^Department of Psychiatry, Korle-Bu Teaching Hospital, Accra, Ghana

## Abstract

Cardiovascular symptoms like chest pain and palpitations are among the commonest reasons for outpatient hospital visits. Physician preoccupation with ruling out sinister cardiological diagnoses often results in a failure to recognise mental health-related disorders, which account for over 40% of such cases, especially among young women. These disorders can independently cause cardiovascular symptoms or worsen preexisting cardiovascular disease, worsening morbidity. The pathophysiology of mental stress-induced myocardial ischaemia involves increased levels of neurotransmitters, as opposed to anatomical obstruction seen in conventional coronary artery disease. This results in a battery of tests (including invasive assessments) which yield normal results, deepening the patient's psychological stress. There is therefore an increased expenditure on healthcare with little assurance of wellness. Detection of these conditions is poorer in developing countries due to limited capacity in appreciating mental health disorders, though over 70% of mental health disorders occur in these countries. Two young ladies with no comorbidities who presented with chest pain and palpitations are reported in this paper. Laboratory investigations and specific cardiology-based tests were normal, but a thorough family and social history revealed underlying mental stresses, corroborated by a mental state examination. A diagnosis of panic disorder was made and once managed, symptoms abated and quality of life improved. We seek to emphasise that mental health disorders are an important cause of cardiovascular symptoms among young adults and must be actively sought by physicians to reduce the associated morbidity, as conventional tests for ischaemia are not useful in their diagnosis. Mental state examination should be done routinely in all clinical assessments to identify patients with subtle signs and improve their clinical outcomes. There must be commitment to build capacity among nonpsychiatrists to reduce the treatment gap.

## 1. Introduction

Cardiovascular disease is the leading cause of mortality worldwide [1], making cardiovascular symptoms a major concern globally. Chest pain and palpitations account for majority of cardiology referrals worldwide [2, 3], with the prevalence of chest pain among primary healthcare patrons in the United States approaching 80% [1, 2]. 50-63% of these patients have no identifiable cause despite extensive cardiological assessment, leading to a diagnosis of noncardiac chest pain (NCCP) [1–4].

Chest pain and palpitations have been described as symptoms of mood and anxiety disorders [5], highlighting the relationship between mental health and cardiovascular disease, particularly among women under age 50 [5]. Failure to identify and address underlying mental health problems results in increased morbidity and hospital costs due to multiple hospital visits with numerous investigations being performed [1, 2]. Consequently, patients are unassured because the absence of positive findings on investigations while they are symptomatic worsens anxiety [2, 3], culminating in a vicious cycle. Some patients may, as a result, be dismissed for making up symptoms. Psychiatric causes of chest pain are often considered diagnoses of exclusion [2], after the “more life threatening” conditions like coronary artery disease (CAD) and cardiac arrhythmias are ruled out.

The Global Burden of Disease Study in 2010 identified that 7.4% of disease is attributed to mental health-related disorders [6]. Majority of mental health services are concentrated in developed countries, though up to 80% of the world's population live in low and lower-middle-income countries [6]. It is therefore estimated that over 70% of individuals who require mental health care do not receive this, creating a significant treatment gap [6–8]. The size of the treatment gap is multifactorial, including poor systems for collaborative care, lack of physician capacity, and patients opting out of being referred to psychiatry for fear of stigmatization [6, 8].

We present two women who reported for cardiology review on account of chest pain, breathlessness, and palpitations. Though cardiology tests returned unremarkable, a mental health assessment showed the presence of significant mental stressors which when addressed, resulted in significant improvement in their symptoms. This seeks to highlight the need to invest in building capacity among nonmental health practitioners to improve overall quality of care in mitigating mental health disorders.

## 2. Case Presentation

Written informed consent was obtained from both patients for anonymized information to be used in this publication.

### 2.1. Case 1

A 40-year-old married female banking executive with no medical history presented with a 5-month history of episodic fast, regular palpitations, associated with chest pain, breathlessness, headaches, and a sense of impending doom. These were often associated with stress and lasted about 3 to 7 minutes. She had an average of one episode a week and experienced significant concern about another episode occurring. She had an average of 5 hours of sleep per night, often feeling unrefreshed on awakening. She often dreamt about work. Over the period, she had generally felt as though “all was not well.” She had no domestic problems but felt stressed because she worked 50-60 hours weekly. The last episode prior to her review was 2 weeks earlier, following which she reported at a primary care facility and was told she had a fast heart rate for unknown reasons as investigations were normal. Unassured, she self-reported for a cardiology review.

Clinical system examination was normal. She looked well, kempt, and calm with no psychomotor agitation. She had normal rate, tone, and volume of speech, which was both coherent and relevant. She described her mood as mildly sad, which she attributed to concerns about her condition. Her affect was however reactive. Her thoughts were preoccupied with concerns about stress at work and her health, although she did not express hopelessness or suicidal ideation. There were no perceptual abnormalities and cognitive assessment revealed no abnormalities. Electrocardiogram (ECG), full blood count, and serum electrolytes were all normal.

She was assessed using the Mini International Neuropsychiatric Interview, and a diagnosis of panic disorder was made based on the International Classification of Diseases, 10^th^ edition (ICD-10) diagnostic criteria. She received psychoeducation on her condition and stress management. She was started on a two-week course of lorazepam 2 mg daily and propranolol 40 mg bd.

On review a week later, she admitted to feeling much better after the initial consultation once she was given a diagnosis and understood what was happening. She felt more relaxed, had a better week at work, and had better quality of sleep. She has not had any further episodes a month after initial consultation. She reports that following psychoeducation, she has appreciated the role she has to play in managing stress to prevent symptoms and therefore feels more empowered to safeguard her own health.

### 2.2. Case 2

A 33-year-old female nurse with no known comorbidities reported a 3-month history of recurrent palpitations, breathlessness, diaphoresis, and a choking sensation. During episodes, she felt helpless and that she was going to die. These episodes were often triggered by stress but would sometimes occur at rest. These symptoms began soon after the passing away of a close relative, which coincided with the termination of her relationship with her fiancé. During this time, she admits experiencing social withdrawal, anhedonia, feelings of guilt and hopelessness, and insomnia. She had visited various health facilities, but all tests done were unremarkable. She was rushed to hospital during an episode while a 48-hour Holter ECG was being recorded as part of her clinical workup.

On assessment, she was dyspneic with a respiratory rate of 32 cycles per minute. All other clinical systems were normal. She was kempt, but apprehensive and restless. Her speech was hesitant and labored, with difficulty completing sentences. She was preoccupied with fears of dying, but did not experience derealization, depersonalization, or other perceptual abnormalities. Venous blood gases showed a respiratory alkalosis (Table 1), consistent with hyperventilation. ECG was normal. Symptoms resolved within an hour. The next day, repeat venous blood gases were normal (Table 1). Echocardiogram and 48-hour Holter monitor were normal.

She was further assessed with the Mini International Neuropsychiatric Interview. A diagnosis of panic disorder with comorbid moderate depressive episode without somatic syndrome was made, based on the International Classification of Diseases, 10^th^ edition (ICD-10) diagnostic criteria. She was discharged on atenolol 50 mg daily and amitriptyline 25 mg nocte. She travelled for 3 years and was unable to have regular medical reviews, resulting in her discontinuing the medications. She however had fewer episodes after changing jobs to a less stressful health center.

On a recent review, she was commenced on oral paroxetine 20 mg nocte following discussions with a psychiatrist and has since been well, with no reported adverse effects. She reports feeling generally better and has had no further episodes after 2 months.

## 3. Discussion

The patients described above had clinical histories that pointed to mental health disorders. This was however missed because of physician focus on the medical aspects of the presentation. A Saudi study showed that capacity in managing mental health disorders is low among various cadres of doctors, with family physicians having the highest level of knowledge among nonpsychiatrists [8]. Capacity building, among both clinicians and researchers, is key to closing the treatment gap and improving overall care and should not be restricted to psychiatrists and psychologists [7] to prevent recurrence of cases like these. The scope of capacity building must be widened because there is still stigma associated with mental health disorders, which prevents patients from willingly attending centralised mental health centres [7, 8]. All clinicians must therefore have an acceptable minimum level of knowledge to assist such patients.

Young adults (mean age = 32.8 years) [1] are the most at risk of anxiety-related psychiatric disorders which may present with cardiovascular symptoms, commonly among women [1, 5, 9, 10]. Reasons include occupational, socioeconomic, and marital/reproductive stressors. The frequency of NCCP diminishes with advancing age [1] due to a reduced effect of these stressors.

Panic disorder accounts for up to 44% of NCCP cases [1, 4], with 75-85% of cases [1] having comorbid mood and anxiety disorders. Physician focus on excluding organic cardiovascular disease like CAD however results in underdiagnosis of mental health disorders as a cause of symptoms [4], as evident in both patients described above. Patients may be told “there is no blockage in your arteries causing the symptoms, so you are fine.” Those suspected to have anxiety as a contributor may be prescribed with short-term benzodiazepines without psychiatric evaluation. Although benzodiazepines are effective for short-term management of anxiety disorders, they are less effective long-term, with adverse effects such as cognitive impairment, dependence, and withdrawal symptoms [11]. Patients may therefore experience temporary relief but frequently relapse.

Mental stress-induced myocardial ischemia (MSIMI) is an independent risk for cardiovascular morbidity [5], with its effects mediated by serotonergic and noradrenergic pathways [9]. Conventional cardiovascular assessments of myocardial ischemia based on anatomical obstruction to coronary blood flow are therefore ineffective in diagnosis [5]. Mental stress causes chronic endothelial dysfunction, resulting in microvascular dysfunction, myocardial ischemia, and an increased risk of stroke [1, 5]. MSIMI can either cause de novo daily angina disproportional to level of activity [2, 10] or aggravate pre-existing CAD, worsening prognosis [5, 10], with frequency of symptoms regarded as a marker of psychological distress [10]. Symptoms occur at lower oxygen demands than seen in CAD [10], reducing quality of life [2, 10].

A biopsychosocial consultation model is helpful [2], remembering that each patient with a biological condition has a psychosocial predisposition influenced by work, friends, and family that informs coping mechanisms, illness behaviour, and perceptions. These interplay intricately to manifest disease. This therefore requires a good social history and mental state examination to offer the best possible care. Success is however predicated on a good physician-patient relationship [2], which allows the patient to open up about details of personal life. Emergency care and investigations must however not be sacrificed for mental health assessments [4]. Patients who present acutely should be investigated to eliminate life-threatening ailments, as done in case 2.

A good mental state examination identifies patients with mental health disorders even without overt symptoms. Although it may be impractical to do a full mental state examination for all patients, relevant aspects can be incorporated in the clinical assessment, especially when the presentation could include mental health problems [12]. This involves observing appearance and behavioral cues that may point to psychological or mental problems. These include psychomotor agitation or retardation and poor eye contact. A patient with a sad or restricted range of expressed affect may also point to psychosocial stress. Patients should routinely be asked about their mood, as well as preoccupations with negative thoughts or worry regarding aspects of their lives or health. Perceptual disorders such as hallucinations, perceptual distortions, derealization, and depersonalization may also be identified.

Up to 75% of patients presenting with chest pain are treated conventionally for myocardial ischemia without evidence of CAD, without exploring mental stress as a cause [1, 2]. This gives credence to the theory of focusing on medical symptoms rather than the entire patient [8]. Cognitive behavioral therapy (CBT) is beneficial in the treatment of such patients [2], though different authors have varied views on the role of selective serotonin receptor inhibitors (SSRIs) [2, 9]. A combination of SSRIs and CBT has however been found to be more effective than monotherapy [13], with education on coping methods and stress management techniques also being useful [5].

## 4. Conclusion

Psychiatric causes of NCCP are a significant cause of morbidity, comparable to CAD with symptoms occurring at lower levels of activity and should be considered early in the differential diagnosis. General practitioners, cardiologists, and psychiatrists must liaise with one another to ensure prompt diagnosis, early referral, and appropriate treatment. There should be investment in continuing medical education programmes to empower other clinicians in recognizing and managing common mental health disorders. Negative results should not lead physicians to discredit symptoms, but rather prompt them to rule out mental health disorders. Coping mechanisms are key in the care of such patients to improve their outcomes and give them a better sense of satisfaction.

## Figures and Tables

**Table 1:** Laboratory test results for case 2.

Parameter	Day 1	Day 2	Reference range
Venous blood gas			
pH	7.55	7.39	7.30-7.40
pO_2_	32	42	35-45 mmHg
pCO_2_	25	36	42-48 mmHg
Electrolytes			
HCO_3_^−^	21.9		22-28 mmol/L
Na^+^	138	138	135-150 mmol/L
K^+^	3.0	4.2	3.5-5.0 mmol/L
Ca^2+^		1.36	1.10-1.35 mmol/L
Mg^2+^		0.70	0.65-1.10 mmol/L
D-dimer		<0.1	<0.5 mg/L

Legend: pO_2_: partial pressure of oxygen; pCO_2_: partial pressure of carbon dioxide; HCO_3_^−^: bicarbonate; Na^+^: sodium; K^+^: potassium; Ca^2+^: calcium; Mg^2+^: magnesium.

## Data Availability

No data were used to support this study.
